# Cross-linguistic patterns of cognitive biases in large language models: a comparative study in English, Hebrew, and Russian

**DOI:** 10.3389/frai.2026.1808549

**Published:** 2026-06-17

**Authors:** Katya Grander, Victoria Mosub, Sigal Kordova

**Affiliations:** 1 Faculty of Industrial Engineering and Technology Management, Holon Institute of Technology, Holon, Israel; 2 Department of Industrial Engineering and Management, Ariel University, Ariel, Israel

**Keywords:** large language models, cognitive biases in LLMs, cross-linguistic LLM performance, machine psychology, LLM-human reasoning comparison, multilingual AI evaluation

## Abstract

Large Language Models (LLMs) are being increasingly incorporated into decision-support systems. Nonetheless, a lack of clarity remains with reference to their reasoning processes, particularly in multilingual contexts. This uncertainty extends to cognitive biases - systematic errors in judgment, similar to those documented in human cognition. Existing research on cognitive biases in LLMs has focused primarily on English-language settings and a limited range of model families, leaving open the question of whether bias manifestations differ across input languages and distinct architectures. The current study investigated the ability of three widely used LLMs (ChatGPT, Claude, and Gemini) to solve cognitive tasks targeting availability heuristics and confirmation bias, comparing their performance to a human control group. The tasks were administered in English, Hebrew, and Russian, representing Germanic, Semitic, and Slavic linguistic contexts. The analytical dataset comprised 2,028 observations: 507 human responses, collected via dedicated online questionnaires, and 1,521 LLM-generated responses, obtained through API interfaces. Statistical analyses implemented Pearson’s Chi-Square tests, post hoc comparisons with Bonferroni correction, logistic regression, and Firth penalized logistic regression to compare correctness patterns across models, tasks, and languages, with human performance serving as a baseline. The results revealed a “cognitive gap”: LLMs consistently outperformed human participants on the rule-based deductive task, yet exhibited bias-mimicking error patterns in heuristic reasoning-based tasks. The observed effects varied significantly across languages and models, challenging the expectation of uniform multilingual performance and suggesting that LLM architecture interacts with linguistic structures in unpredictable ways. Overall, the findings indicate that cognitive bias expression in LLM outputs is not merely a technical constraint but a language-dependent phenomenon with practical implications for deployment in multilingual environments. The study emphasizes the need for cross-linguistic evaluation when assessing the reliability of LLM-based decision-support systems, particularly in domains where biased reasoning may affect judgment and decision quality.

## 1 Introduction

In recent years, Large Language Models (LLMs) have been widely adopted across commercial, professional, and public domains. These transformer-based architectures, trained on vast multilingual datasets, have demonstrated unprecedented capabilities in processing natural language, enabling them to parse complex linguistic structures and maintain coherent discourse during interactions with their users.

However, despite such impressive proficiency, growing empirical evidence indicates that LLMs may reproduce forms of cognitive biases, similar to those documented in human cognition (Azaria, 2023; Berberette et al., 2024; Castello et al., 2024; Chen et al., 2025; Echterhoff et al., 2024; Santtila et al., 2026) i.e., systematic deviations from normative reasoning patterns in judgment and decision-making processes (Tversky and Kahneman, 1974). This phenomenon carries significant theoretical and practical implications. Since users increasingly regard LLMs as authoritative information sources (Eigner and Händler, 2024), biased outputs can influence judgment and distort decision-making processes, thereby posing risks to the quality and reliability of decisions in sensitive domains such as healthcare, education, or finance - elevating the presence of cognitive biases in AI systems from a mere technical issue to an ethical concern.

Bias manifestation in LLMs’ outputs can be attributed, in part, to the human-derived nature of the models’ architecture and training datasets, which may contain inherent biases or skew the representation of certain phenomena (Choudhary, 2025). Additionally, given the linguistic variation in training corpora, bias expression and its associated effects may differ systematically across languages with distinct syntactic or cultural frameworks (Castello et al., 2024).

The objective of this study was to investigate the presence of cognitive biases in the outputs of three popular Large Language models—ChatGPT, Gemini, and Claude—by addressing cross-linguistic aspects of LLMs’ performance and examining whether the models’ susceptibility to cognitive biases varies across input languages. The analysis encompassed three typologically distinct natural languages (Škrlj and Pollak, 2019; Fridman et al., 2023)—English, Hebrew, and Russian—thereby extending previous findings beyond Italic and Germanic languages (Castello et al., 2024) to include the Semitic and Slavic branches.

The study, therefore, pursues three main research questions:

1. To what extent does availability heuristic manifest in the outputs of ChatGPT, Gemini, and Claude?
2. To what extent does confirmation bias manifest in these same models?
3. How do these manifestations differ across input languages (English, Hebrew, Russian)?

While the motivation for the investigation emerges from the following imperatives:

### 1.1 Theoretical contribution

Previous research reviewed for this study’s theoretical foundation focused primarily on a single LLM family (ChatGPT) and was largely constrained to English contexts. By examining availability heuristic and confirmation bias across three models (ChatGPT, Claude, Gemini) and three languages (English, Hebrew, Russian), this study expands theoretical understanding of bias manifestation in artificial systems. It contributes to the emerging field of “machine psychology,” which applies psychological frameworks to AI behavior (Hagendorff et al., 2024).

### 1.2 Practical relevance

LLMs are increasingly used in decision-making processes in domains such as medicine, education, and law (Berthet, 2022; Castello et al., 2024; Santtila et al., 2026). If these systems exhibit response patterns similar to those observed in human reasoning, their outputs could influence critical choices with quite tangible consequences. By comparing LLM outputs to human baselines (as represented in this study), this research identifies conditions under which models may mimic human fallacies rather than exhibit rational reasoning. Moreover, through a cross-linguistic analysis of cognitive bias expression across three language families, this research suggests how linguistic context may affect model behavior, emphasizing the importance of fairness evaluation in multilingual AI implementation.

### 1.3 Technology management and risk assessment aspects

This study provides technology and product managers with new insights into LLMs’ performance across different languages and contexts, contributing to the design of risk assessment frameworks that account for linguistic variability in AI performance.

### 1.4 Social and ethical considerations

This research highlights the imperative for critical and informed use of AI tools such as Large Language Models, recognizing that cognitive biases are not uniquely human phenomena but may also emerge within sophisticated automated systems.

## 2 Background

### 2.1 Cognitive biases and human reasoning

Life decisions are often made in the absence of complete information, with little knowledge of the unforeseen obstacles that may arise. Conscious (or rational) decision-making under uncertainty is based on two main characteristics of possible outcomes: their desirability or undesirability and the probability of their occurrence. In contrast, rational choice theory involves developing methods to ensure the selection of an optimal solution to a given problem (Korteling and Toet, 2020). According to this approach, a rational decision-maker should first consider all available information, the probabilities of events, and the costs and benefits of possible outcomes, and only then choose the best option. Nevertheless, many human decisions are not optimal and tend to exhibit consistent systematic deviations in judgment and decision-making processes (Tversky and Kahneman, 1974; Wason and Shapiro, 1971).

The term “cognitive biases,” introduced by Tversky and Kahneman (1973, 1974), describes these deviations. Cognitive biases are unconscious, consistent errors in thought that emerge as individuals process and interpret information from their surroundings, ultimately shaping their decisions and evaluations. Extensive research indicates that such biases are widespread across all areas of society, including fields of paramount importance. In management and strategic contexts, the existence of biases, such as availability, hindsight, and overconfidence, often leads to suboptimal decisions (Bazerman and Moore, 2012; Berthet, 2022), while in medicine, a review of 213 studies confirmed presence of biases in 68% of the cases, with availability heuristic and framing bias among the most prevalent (Blumenthal-Barby and Krieger, 2015). Legal research highlights the role of confirmation and anchoring biases in judicial reasoning (Bystranowski et al., 2021; Teichman et al., 2023), whereas financial studies emphasize overconfidence and the disposition effect, rooted in loss aversion (Berthet, 2022). Even science is not immune, as researchers themselves may fall prey to confirmation bias despite methodological safeguards (Pinto, 2023). Overall, evidence indicates that cognitive biases are systematic, pervasive, and influential across decision-making domains, underscoring the importance of critical thinking, awareness, and data-driven approaches to mitigate their impact. Moreover, cognitive biases tend to persist even when individuals lack sufficient information or recognize their own susceptibility, often reinforcing unwarranted confidence in their judgments (Korteling and Toet, 2020).

Within this extensive field, the current research narrows its focus to two biases that we consider particularly relevant to information retrieval and evaluation: confirmation bias, which reflects the tendency to search for or to interpret the evidence in a way that is predisposed in favor of existing beliefs, expectations, or hypotheses (Nickerson, 1998); and availability heuristic, which refers to the mental shortcut by which individuals estimate the likelihood of an event based on how readily similar examples or experiences come to mind, often skewed by vivid or recent experiences (Tversky and Kahneman, 1974).

### 2.2 Large language models and their design principles

Language is a vital tool for expression and communication that humans acquire after birth and continue to use throughout their lives. Machines, however, do not possess the ability to understand and speak human language. Therefore, a long-standing scientific challenge has existed for machines to achieve human-like reading, writing, and communication skills (Raiaan et al., 2024). Advances in deep learning approaches, availability of cutting-edge computing resources, and access to extensive training data have contributed to overcome this challenge. This has led to the creation of Large Language Models (LLMs), designed to understand, process, and generate human-language outputs. Their applications span a wide range of domains, including content creation, automated customer service, programming assistance, and more specialized tasks such as supporting medical diagnoses or analyzing legal documents. The versatility and scope of LLM applications have made them an essential tool in the modern digital landscape.

This study focuses on three popular Large Language Model families—ChatGPT, Gemini, and Claude. Certain studies (Sobo et al., 2025) argue that these families represent, in essence, different philosophical approaches to LLM development:

ChatGPT or Chat Generative Pre-Trained Transformer (developed by OpenAI), uses the Reinforcement Learning from Human Feedback (RLHF) technique to generate text that simulates human writing style in response to user instructions. Its development focuses on creating a versatile conversational agent capable of understanding and generating coherent, contextually relevant responses to assist with a wide range of tasks, from text composition to answering complex questions that involve logical inference.

Gemini (formerly known as Bard, developed by Google) is a multi-modal model, in which the pre-training dataset utilizes data from internet documents, books, and code, including text, images, audio, and video (Anil et al., 2023). This approach aims to create a more comprehensive understanding of information and to allow the model to perform tasks that require integrating text and visual information.

Claude (developed by Anthropic) is built on the philosophical principles of Constitutional AI, focusing on safety and alignment with human values. Anthropic’s goal was to develop a model with a lower likelihood of producing harmful and offensive outputs, with emphasis on meeting ethical requirements.

### 2.3 Cognitive biases in large language models

Alongside their exceptional performance in understanding and processing natural language, Large Language Models face two significant challenges that were previously used to describe human behaviors, but have recently been adapted to the LLM domain to define machine functional deficits (Castello et al., 2024). This adaptation was necessary because, although Large Language Models do not possess true cognition, they can nonetheless exhibit traits that functionally mirror human cognitive tendencies.

The first challenge stems from the fact that, despite their ability to produce coherent outputs that simulate human language and logic, Large Language Models are, in essence, unable to distinguish between truth and falsehood and therefore may confidently present fabricated answers as if they were true (Roberts et al., 2024). This behavior is termed “hallucination.” In the context of LLMs, hallucination refers to the creation of content or outputs that exhibit grammatical correctness, fluency, and authenticity but do not correspond to the original input (lack faithfulness) or do not align with factual accuracy (lack factuality) (Ye et al., 2024).

An additional challenge, which is the subject of this study, arises due to the fact that Large Language Models are trained on vast amounts of data from diverse human-influenced sources, including online textual corpora such as websites and comparable digital repositories. As a result, the models may inherit offensive, stereotypical, misleading, or discriminatory behaviors. These behaviors are an expression of what is commonly called “bias” (Lin et al., 2024).

Bias, in the context of Large Language Models, is defined as the presence of systematic distortions, attribution errors, or factual distortions that cause a preference for specific groups or ideas, the perpetuation of stereotypes, or the generation of inaccurate conclusions derived from learned patterns (Ferrara, 2023a). While the training dataset and its quality are believed to be the most significant sources of bias, regardless of its type (Mondal and Lipizzi, 2024), additional areas where bias can affect Large Language Models include data collection, algorithm design, and human interpretation (Ferrara, 2023b).

Biases in Large Language Models are expressed in various forms. Demographic biases emerge when the training data represent certain demographic groups, thereby leading the model to exhibit biased behavior toward gender, race, ethnicity, or other social groups (Zubiaga, 2024). Cultural biases occur when LLMs learn and perpetuate stereotypes about various cultures from their training data, potentially leading the model to produce outputs that reinforce or exacerbate existing cultural prejudices. Temporal biases arise when training data is limited to specific time periods or is otherwise unrepresentative, which may prevent the model from fully understanding the historical context. Since most internet data is in English and a limited set of other dominant languages, Large Language Models tend to exhibit uneven performance and limited coverage for languages with limited digital presence or local dialects, thereby exhibiting linguistic biases. Similarly, models may learn and propagate ideological and political biases, leading to outputs that favor certain political viewpoints or ideologies (Ferrara, 2023a).

Given that cognitive biases are intrinsic to human thought processes (Tversky and Kahneman, 1974), it is reasonable to assume that these biases will also be reflected in the models’ training data, architecture, algorithms, calibration methods, and interactions with users (Choudhary, 2025). In other words, Large Language Models may inherit and perpetuate cognitive biases, which will be expressed in the outputs they produce.

Recent studies on generative AI apply the “machine psychology” method, which positions Large Language Models, through their chatbot interfaces, as subjects in psychological tests designed initially to study non-normative human behaviors (Hagendorff et al., 2024). Using this approach, examination of cognitive biases in LLMs relies, in one way or another, on tasks proposed by Kahneman and Tversky (Tversky and Kahneman, 1974) for testing cognitive biases in humans (Azaria, 2023; Chen et al., 2025).

Previous research confirmed LLMs’ susceptibility to at least availability, overconfidence, representativeness, framing, and confirmation biases (Berberette et al., 2024; Chen et al., 2025; Kliegr et al., 2021; Santtila et al., 2026). Yet, despite this extensive body of research, most of it has focused primarily on models from the GPT and Llama families (Sumita et al., 2025). The current study aimed to expand the existing findings and examine how confirmation bias and availability heuristic are expressed across three families of Large Language Models: ChatGPT, Gemini, and Claude.

### 2.4 Cross-linguistic analysis of cognitive biases in LLMs

As mentioned above, cultural, linguistic, and economic biases in Large Language Models stem primarily from their internet-based training data, which is heavily skewed toward specific cultures, languages, and economic perspectives (Qu and Wang, 2024). This happens because the training process requires extensive text corpora. However, these corpora are unevenly distributed across different languages. One reason is that countries with high economic status typically have more extensive digital footprints, as their citizens are more likely to access the internet and contribute content. As a result, LLMs appear to perform adequately in common languages such as English, German, and French but poorly in low-resource languages. For example, research shows that some LLMs cannot understand the true meaning of slang terms with specific cultural backgrounds, such as Chinese dialects (Li et al., 2025).

Although a substantial body of research has examined the cross-linguistic performance of Large Language Models (Hu et al., 2025; Rathje et al., 2024; Qin et al., 2025; Sozen Yanik et al., 2025), relatively little attention has been given to how cognitive biases manifest across different input languages. A previous study examined the presence of three cognitive biases—availability, representativeness, and framing—through linguistic comparisons between the performance of ChatGPT-3.5 and ChatGPT-4 in three languages—English, Italian, and Spanish—and found that ChatGPT-3.5 showed statistically significant differences in performance across these languages (Castello et al., 2024). It is important to note that Spanish and Italian belong to the same language branch, i.e., Romance, and that all three languages have a very high digital presence. Additionally, the research only compared performance of Large Language Models from the same family—the GPT family. As of late 2025, we did not find any additional studies on linguistics and cognitive biases in Large Language Models.

The current study addresses these limitations by examining three language branches—Germanic (English), Slavic (Russian), and Semitic (Hebrew)—each with a high level of digital presence but with different levels of representation by orders of magnitude. The English language has 1.5 billion speakers worldwide (Ethnologue, 2025), and Russian has approximately 253 million speakers (Ethnologue, 2025), while the number of Hebrew speakers is estimated at approximately 9 million worldwide (Turetzky et al., 2024). The aim of this research was to examine whether, assuming significant differences in training corpus sizes, as well as linguistic, cultural, historical, and economic factors, the performance of Large Language Models in the context of cognitive biases across these three languages will differ (see Table 1).

**Table 1:** Summary of related works.

Study name, year	Tested cognitive biases	Models	Human baseline	Cross-linguistic focus	Languages
Jones and Steinhardt (2022)	Framing, anchoring, availability, attribute substitution	OpenAI Codex, Salesforce CodeGen, GPT-3	Comparison to established findings	No	English
Azaria (2023)	Framing, intuition bias, availability, iterative reasoning	ChatGPT	Comparison to established findings	No	English
Hagendorff et al. (2023)	Intuitive reasoning errors, cognitive reflection effects, human-like cognitive errors	GPT family from GPT-1 to ChatGPT-4	Comparison to established findings	No	English
Lin and Ng (2023)	Availability, confirmation, anchoring	GPT-2, GPT-Neo, GPT-J, OPT, BLOOM, GPT-3	Comparison to established findings	No	English
Berberette et al. (2024)	Availability, cognitive dissonance-related framing	Multiple	No	No	English
Macmillan-Scott and Musolesi (2024)	Confirmation, conditional probability, gambler’s fallacy, endowment effect, conjunction fallacy, representativeness effect, misconception of chance	GPT-3.5, GPT-4, Bard, Claude 2, Llama 2 7B, Llama 2 13B, Llama 2 70B	Comparison to established findings	No	English
Lampinen et al. (2024)	Content effects, belief bias	Chinchilla, PaLM 2-M, PaLM 2-L, Flan-PaLM 2, GPT-3.5-turbo-instruct	Yes	No	English
Castello et al. (2024)	Availability, framing effect, representativeness	ChatGPT 3.5, ChatGPT 4	Yes	Yes	English, Spanish, Italian
Echterhoff et al. (2024)	Anchoring, framing effect, status quo bias, group attribution, primacy	GPT-3.5-turbo, GPT-4, Llama 2 7B, Llama 2 13B	Comparison to established findings	No	English
Schmidgall et al. (2024)	Information bias, availability, confirmation, overconfidence, anchoring, order bias, premature closure bias	GPT-3.5, GPT-4, Meditron-70B, Llama 2-7B, Llama 2-13B, Llama 2-70B	Comparison to established findings	No	English
Chen et al. (2024)	Priming effect	GPT-3.5, GPT-4o, LLaMA2-13B, LLaMA2-70B	Comparison to established findings	No	English
Chen et al. (2025)	Multiple	GPT-3.5, GPT-4	Comparison to established findings	No	English
Sumita et al. (2025)	Multiple	GPT-3.5, GPT-4	No	No	English
Santtila et al. (2026)	Outcome bias, anchoring bias, anti-inference bias	GPT-4o, GPT-o3-mini, DeepSeek-R1, Claude-Sonnet-4, Llama-3.3-70B, Mistral-Large-2411, Qwen-235B-A22B	Replicates data from prev. Study (Teichman et al., 2023)	No	English
Abe et al. (2026)	Confirmation, matching bias	gpt-oss-20B, gpt-oss-120B, Qwen 3 14B, Qwen 3 32B, Gemma 3 4B, Gemma 3 12B, Gemma 3 27B, Llama 3.3 70B, OLMo 2 32B	Comparison to established findings	No	English
Current study (2026)	Availability, confirmation	Claude, Gemini, ChatGPT	Yes	Yes	English, Hebrew, Russian

## 3 Materials and methods

This study employed an experimental research design with manipulated variables across two primary investigative domains: the assessment of cognitive biases (availability heuristic and confirmation bias) in Large Language Models compared to a human control group, and the cross-lingual evaluation of models’ susceptibility to cognitive biases. The research implemented the “machine psychology” approach (Hagendorff et al., 2024) to evaluate LLMs’ responses by using psychological tasks traditionally designed to measure cognitive biases in humans, while the human control group served as a baseline. The outputs of the language models were examined according to the methodology proposed by Macmillan-Scott and Musolesi (2024). Using their method, a query was presented to the LLMs, and their responses were examined based on four parameters: “correct”/“incorrect” and “human-like”/“non-human-like.” “Correct” indicated an answer that accurately addressed the question, while “incorrect” indicated an inaccurate response. “Human-like” and “non-human-like” were defined according to the performance of the human baseline.

### 3.1 Theoretical foundation

#### 3.1.1 Availability heuristic

The examination of the availability heuristic was built on a combination of two previous studies. Firstly, it drew inspiration from the classical task proposed by Tversky and Kahneman—“Word Frequency Test” (1974). The essence of the “Word Frequency Test,” which was originally designed to test the availability heuristic in humans, is explained in Tversky and Kahneman’s article “Judgment under Uncertainty: Heuristics and Biases” (1974): “Suppose one samples a word (of three letters or more) at random from an English text. Is it more likely that the word starts with r or that r is the third letter? People approach this problem by recalling words that begin with r (road) and words that have r in the third position (car) and then assessing the relative frequency by the ease with which words of the two types come to mind. Because it is much easier to search for words by their first letter than by their third letter, most people judge words that begin with a given consonant to be more numerous than words in which the same consonant appears in the third position. They do so even for consonants, such as r or k, that are more frequent in the third position than in the first”.

*Original word frequency test*: Consider the letter R. Is R more likely to appear in: the first position; the third position? Check one (Tversky and Kahneman, 1974).

Secondly, this work built on Castello et al. (2024) research that investigated the presence of availability heuristics in GPT outputs across English, Spanish, and Italian by creating a Modified Word Frequency Test. In the experiment, the same prompt was administered word-for-word to both humans and LLMs: “Based on your knowledge, which of the two words are more common: “yes” or “no”?” (Castello et al., 2024).

#### 3.1.2 Confirmation bias

The examination of confirmation bias in Large Language Models outputs employed a modified version of the well-known “Wason’s Selection Task” (Wason, 1968). In the original task, participants were presented with a deck of cards, each with a letter on one side and a number on the other. Four cards were randomly selected from the deck and placed in front of each participant. The participants were then presented with the following rule: “If there is a D on one side of a card, then there is a 3 on the other side.” The participants’ task was to select only those cards that, if turned over, would show whether the rule is correct or incorrect for the four cards they received. As a result, participants tended to choose either the D card or the D and 3 cards together, but rarely the D and 7 cards (the correct answer). However, if the letter D appeared on the other side of the 7, the rule would be incorrect. Wason attributed participants’ errors in this selection task to confirmation bias, which led subjects to search specifically for cards that confirmed the rule. Meanwhile, they ignored cards that might refute it.

*Original Wason Selection Task (1968)*: You are presented with a set of four cards placed on a table, each with a letter on one side and a number on the other. The cards are: Card: D; Card: K; Card: 3; Card: 7. The rule is: If there is a D on one side of the card, the number 3 appears on its other side.

Which card(s) should you turn over to check that the rule is maintained?

In Wason’s original research, a very low percentage of subjects found the correct solution. In addition to confirmation bias, the low success rate was attributed to the test question’s abstractness (Wason and Shapiro, 1971). Wason and Shapiro showed that when the task is formulated thematically (thematic task), subjects’ performance improves significantly, leading to the development of a thematically framed version of the selection task:

*Thematic Wason Selection Task (1971)*: You are presented with a set of four cards placed on a table, each with a city name on one side and a picture of a vehicle on the other. The cards are: Card: Manchester; Card: Leeds; Card: Car; Card: Train. The rule is: Every time I go to Manchester, I travel by car. Which card(s) should you turn over to check that the rule is maintained?

To address these limitations of abstract task designs, the current research employed a more practical version of a task. The task was additionally modified due to the high recognizability of the classic experiments, while the logic behind it remained unchanged. The modification was inspired by methodology proposed in a previous study (Lampinen et al., 2024).

### 3.2 Research instruments

Based on this theoretical foundation, the current study implemented three cognitive tasks with condition-specific versions for the human control group and the Large Language Models:

*Original Word Frequency Test (OWFT)*: When randomly selecting an English word that is at least three letters long, which scenario is more likely?

1. The word begins with the letter R (e.g., “red,” “rainbow”).
2. The letter R appears as the third letter in the word (e.g., “border,” “cork”).
3. Both scenarios are equally likely.

*Modified Word Frequency Test (MWFT)*: Which English word do you believe is more common?

1. Yes. 2. No. 3. Both are equally common.”

*Modified Wason Selection Task (MWST)*: “An airline check-in agent at John F. Kennedy International Airport must verify passenger documents according to the following rule: “If passengers are flying outside the US, they must present an international passport.” Four passengers approach the agent: Passenger 1: is flying to London; Passenger 2: is flying to Boston; Passenger 3: presents a passport; Passenger 4: presents an ID card. Which action(s) should the agent take to ensure compliance with the rule?

1. Check that the passenger flying to London presented their passport.
2. Check that the passenger flying to Boston presented their ID card.
3. Check that the passenger presenting their passport is flying abroad.
4. Check that the passenger presenting their ID card is taking a domestic flight.”

Human control group received the tasks as a part of an anonymous questionnaire in the research language of their choice, selecting from English, Hebrew or Russian. The two Word Frequency tasks were presented as single-select questions, while the Modified Wason Selection Task was designed as a multiple-select question. Since the questionnaire consisted of single-item measures, internal consistency evaluation was not applicable. Therefore, to assess the clarity and relevance of the questionnaire, the inter-rater reliability was evaluated using Krippendorff’s Alpha. The evaluation was conducted by three independent judges and resulted in acceptable agreement (*α* = 0.717) for clarity and good agreement (*α* = 0.812) for relevance (Krippendorff 2004 threshold).

The LLMs were tested via an API interface. Since both Word Frequency Tests and Wason Selection Task are classical instruments in cognitive psychology, and because “yes” and “no” are unique words, whose frequencies are readily verifiable through online search or recognized from training data, an explicit instruction was prepended to each of the three tasks: “Do not use internet or any knowledge of benchmark tasks, well-known puzzles, or psychological instruments. Treat this as an original, previously unseen item. Base your explanation on general reasoning.”

Each prompt therefore combined the instruction and the task into a single text item. This adjustment was intended to encourage reasoning-based responses rather than simple retrieval from searchable results or surface-level pattern matching. Apart from the instruction prefix, the tasks were identical for models and human participants.

To preserve semantic and pragmatic equivalence across the research languages, the tasks and the instruction were independently translated from English to Hebrew and Russian by two bilingual researchers. The translated versions were then back-translated to English by a native speaker unfamiliar with the study. Any mismatches between translations were resolved through discussion to ensure comparability across languages.

### 3.3 Response correctness criteria

To establish the foundation for “correct”/“incorrect” responses, regarding the frequency of the words “Yes” and “No” in the English language, “Да” and “Нет” in Russian, and “כן” and “לא” in Hebrew, three text corpora were analyzed—one for each language:

- *English [Yes/No]*: Based on the Corpus of News on the Web (NOW), containing over 20 billion English words from 20 dialects, “no” occurs 26,884,733 times, and “yes” occurs 2,024,428 times. A one-tailed Z-test (H₀: p(no) = 0.5; H₁: p(no) > 0.5) showed a significant difference (*z* ≈ 4.63, *p* < 0.001), confirming that “no” appears significantly more often than “yes.”
- *Russian [Да/Нет]*: Based on the Russian National Corpus (over 2 billion tokens), “нет” (“no”) occurs 545,147 times, and “да” (“yes”) occurs 679,307 times. A one-tailed *Z*-test (H₀: p(да) = 0.5; H₁: p(да) > 0.5) showed a significant difference (*z* ≈ 174.75, *p* < 0.001), confirming that “да” appears significantly more often than “нет.”
- *Hebrew [לא/כן]*: Based on a corpus compiled by the National Institute for Testing and Evaluation [1,087,140 words “לא” (“no”) occurs 14,550 times, and “כן” (“yes”) occurs 1,002 times]. A one-tailed *Z*-test (H₀: p()לא = 0.5; H₁: p()לא > 0.5) showed a significant difference (*z* ≈ 108.64, *p* < 0.001), confirming that “לא” appears significantly more often than “כן.”

The responses regarding the position of the letters “R” (English), “Р” (Russian), and “ר” (Hebrew) were classified as “correct/ incorrect” according to the following empirical evidence:

- *English[R]*: Based on Tversky and Kahneman’s research (1974) “R” appears more frequently in the third position
- *Russian[Р]*: An analysis of 5,891 words containing “Р” showed that 1,115 words begin with “Р” and “Р” is the third letter in 1,699 words. The Z-test confirmed that “Р” appears significantly more frequently as the third letter in the word (z ≈ 11.01, p < 0.001)
- *Hebrew[ר]*: An analysis of 19,196 words revealed that 814 words begin with “ר” and in 1,823 words “ר” is the third letter. The Z-test confirmed that “ר” appears significantly more frequently as a third letter (z ≈ 19.65, p < 0.001)

### 3.4 Research procedure

The study was conducted in two main stages:

*Stage I—human participants survey*: upon recruitment, participants received a brief research description and links to online questionnaires in three languages. They were free to select a survey corresponding to their primary language of use (English, Hebrew, or Russian). Each questionnaire contained 11 demographic questions and three control questions, based on the three cognitive tasks (OWFT, MWFT, MWST). The objective of the control questions was to serve as a baseline for evaluating the “human-likeness” (Macmillan-Scott and Musolesi, 2024) of the models’ outputs obtained in Stage II. Following the completion of the questionnaire, responses to control questions were marked as “TRUE” or “FALSE,” based on the empirical basis provided by the response correctness analyses:

- For the *Modified Word Frequency Test*, responses were coded as “TRUE” (correct) when participants selected “No” in English, “Да” in Russian, and “לא” in Hebrew; selections of “Yes”/ “כן”/ “Нет” or “Both” were coded as “FALSE” (incorrect).
- For the *Original Word Frequency Test*, responses were coded as “TRUE” (correct) when the letter R (or its language-specific equivalent) was identified as more likely to occur in the third position, and as “FALSE” (incorrect) when “R” in the first position or the “Both” options were selected.
- For the *Modified Wason Selection Task*, the “TRUE” (correct) and “FALSE” (incorrect) criteria were set according to the logic presented in the original tasks (Wason, 1968; Wason and Shapiro, 1971).

**Figure 1:**
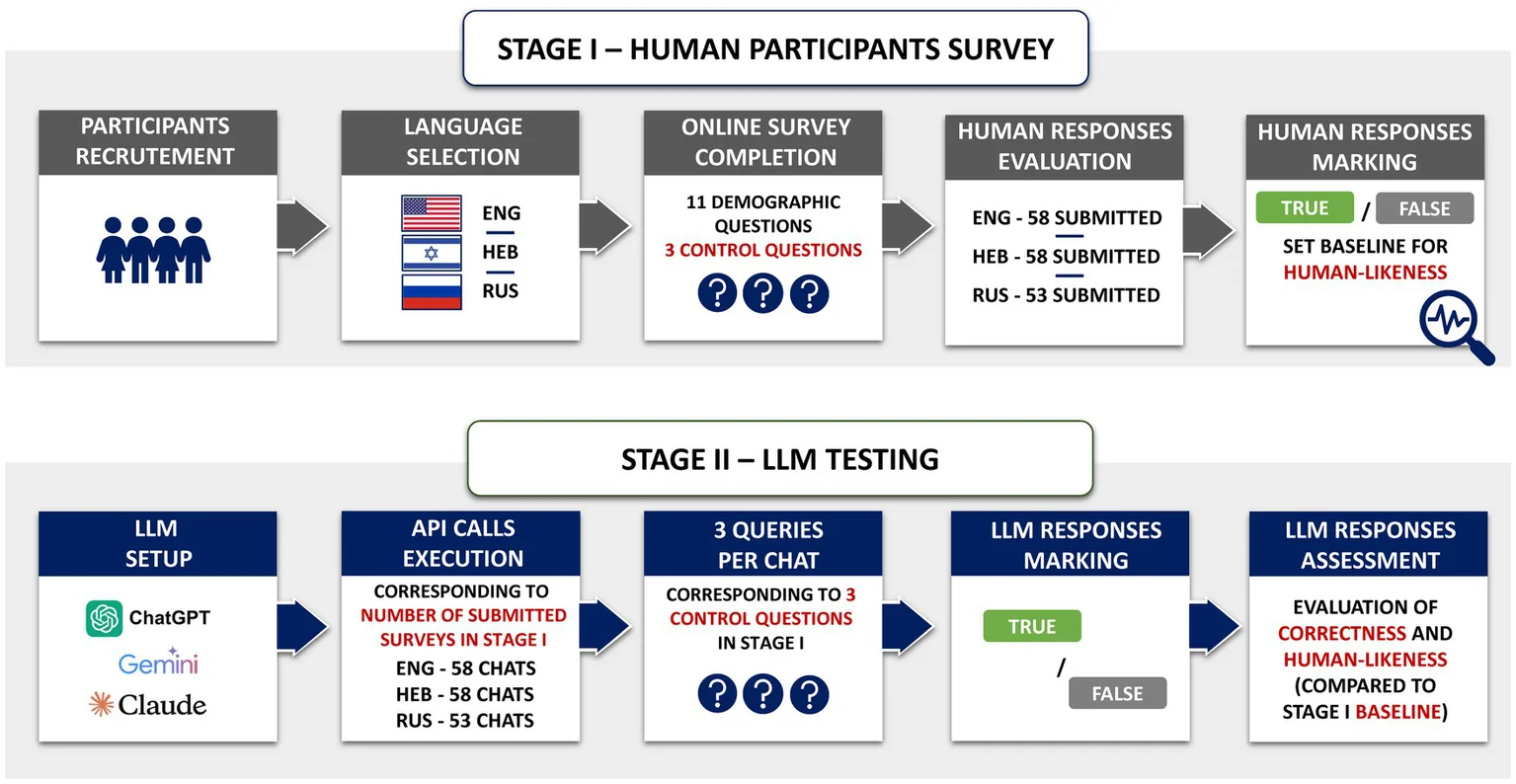
Research stages workflow.

*Stage II—LLM testing*: ChatGPT, Gemini, and Claude were examined across the three research languages (English, Hebrew, and Russian). To enable effective comparative evaluation, multiple independent chats were opened via the API interface for each model and language, corresponding to the number of human participants tested in that language (58 in English and Hebrew, and 53 in Russian). This part of the experiment involved three independent queries, each corresponding to a relevant control question in Stage I. Model responses were evaluated based on “correctness” and “human-likeness” criteria “(Macmillan-Scott and Musolesi, 2024) set in Stage I (see Figure 1).

### 3.5 Participants

#### 3.5.1 Human control group

Non-probabilistic convenience sampling was conducted for the human participants. The participants were recruited through personal outreach, Facebook groups, dedicated forums, and the SurveySwap platform. Efforts were made to diversify participant composition as much as possible across age, gender, and professional or academic background within each linguistic group to ensure the representativeness of the sample. Participation required fluency in at least one of the research languages. The goal was to recruit 50 participants in each language. Ultimately, the number of participants reached 170. One participant was excluded from the research due to failure to provide coherent answers to the research questions. The final research population consisted of 169 individuals (58 in English and Hebrew, and 53 in Russian).

Participants’ demographics showed a balanced representation across adult age groups, with the most significant proportions in the 36–45 (33.7%) and 46–60 (31.2%) age groups. The sample was predominantly female (62.1%), and education levels were overall high: 55.9% held a bachelor’s degree, 27.2% a master’s, and 5.9% a doctorate, while 15.9% reported no higher education. Participants represented diverse professions, with the most common fields being Technology/Computing (31.9%) and Engineering/Exact Sciences (12.4%).

Regarding LLM usage, 88.8% reported using them for less than 2 years. Nearly half (46.2%) recounted daily use, with ChatGPT as the dominant platform (82.2%) and English as the primary language of interaction (66.3%). Approximately one-third (32.0%) stated that they utilized LLMs for both personal and professional contexts. Self-rated digital proficiency was generally high: 25.4% very high, 41.4% high, and 29.6% moderate, with only 3.6% rating themselves low or very low.

#### 3.5.2 Large language models

The research focused on three LLM families—GPT, Claude, and Gemini, examining the versions corresponding to default chatbot interfaces for free LLM users. The tested models were: Chat-GPT-4o (model ID: gpt-4o-2024-11-20), Claude 4 Sonnet (model ID: claude-sonnet-4-20250514), and Gemini 2.5 Flash (model ID: gemini-2.5-flash). All model runs were performed in August 2025.

### 3.6 Model configuration and prompting

To reduce the risk of human error and potential inconsistencies in manual prompt entry and response collection, all LLMs were accessed via their respective official APIs rather than chatbot interfaces: the OpenAI Chat Completions API for ChatGPT-4o, the Anthropic Messages API for Claude 4 Sonnet, and the Google Generative AI API for Gemini 2.5 Flash. Temperature was explicitly set to 1.0 to allow for natural variability in model responses across repeated interactions. The maximum generation limit was set to 8,192 tokens across all models to standardize output constraints and reduce the risk of truncation. No system-level prompts were specified beyond the task instruction and all sessions used default API configurations unless otherwise stated. This approach was intended to approximate the models’ typical behavior and minimize the influence of researcher-imposed settings. The experimental setup implemented a zero-shot, single-problem design to reduce the possibility of priming bias (Chen et al., 2024). Each query was executed as a stateless API session to maintain independence between observations and minimize potential cross-contamination of items. All queries originated from a standardized dataset and were executed through independent, model-specific Python scripts.

### 3.7 Data collection and preprocessing

The analytical dataset included 2,028 observations: 507 human responses (from 169 participants across three tasks) and 1,521 LLM-generated responses (169 iterations for each of the three models across the same three tasks). Human responses were collected via language-specific Google Forms and recorded in Google Spreadsheets, while LLM outputs were collected via API, stored in YAML format and then converted into tabular form using a Python preprocessing script with pandas library (v2.3.1). The dataset was then manually organized in a multi-sheet Microsoft Excel workbook to support analysis across tasks, languages, and response sources. Task-specific sheets recorded responses for a single task within one language, with each row representing a single response and each column representing a response source (Human, ChatGPT, Claude, or Gemini). Model-specific sheets recorded each model’s performance across the three language conditions (English, Hebrew, and Russian), while summary sheets tracked overall LLM performance patterns by language. All responses were coded “TRUE”/“FALSE” according to criteria described in Section 3.4.

### 3.8 Ethical considerations

This study received ethical approval from the Ethics Committee for Non-Clinical Studies at Ariel University. The ethics committee confirmed that the study upholds the institutional procedure for approval of studies that are not medical experiments involving human participants. All human participants in the control group provided their written informed consent prior to participation in the study. The study design ensured complete participant anonymity, and no personally identifiable information was collected or stored throughout the research process.

### 3.9 Statistical analysis

Statistical analyses of the dataset were conducted in R (version 4.5.2), a language and environment for statistical computing (R Foundation for Statistical Computing, Vienna, Austria). The analyses utilized the following R packages: tidyverse for data wrangling and visualization, rstatix for statistical testing, vcd for categorical data analysis, and readxl for data import. The analyses employed Pearson’s Chi-Square test for independence, followed by *post hoc* comparisons, where the Bonferroni correction was applied to all *p*-values to address the risk of multiple-comparison false positives. Logistic regression analyses were conducted to further validate the observed patterns. Finally, Firth penalized logistic regression was employed to address imbalanced outcome distributions and potential complete or quasi-complete separation effects (Firth, 1993).

## 4 Results

### 4.1 Task-level chi-square analysis

As an initial step, for each cognitive task, Pearson’s Chi-Square tests were conducted to assess the association between the response source (Human, ChatGPT, Claude, Gemini) and response correctness across the languages (English, Hebrew, and Russian).

Effect sizes for Chi-Square tests were interpreted according to Cohen’s (1988) benchmarks: small ≥ 0.10, moderate ≥ 0.30, large ≥ 0.50 (see Table 2).

**Table 2:** Contingency table of response correctness by task, source, and language.

Source	English			Hebrew			Russian		
	Incorrect	Correct	%Correct	Incorrect	Correct	%Correct	Incorrect	Correct	%Correct
Modified word frequency test									
Human	42	16	27.6%	19	39	67.2%	43	10	18.9%
ChatGPT	56	2	3.5%	9	49	84.5%	4	49	92.5%
Claude	47	11	18.9%	0	58	100%	0	53	100%
Gemini	38	20	34.5%	1	57	98.3%	37	16	27.2%
Original word frequency test									
Human	44	14	24.1%	44	14	24.1%	34	19	35.8%
ChatGPT	24	34	58.6%	17	41	70.6%	3	50	94.3%
Claude	16	42	72.4%	16	42	72.4%	30	23	43.4%
Gemini	3	55	94.8%	0	58	100.0%	16	37	69.8%
Wason selection task									
Human	47	11	19.0%	41	17	29.3%	27	26	49.1%
ChatGPT	9	49	84.9%	7	51	87.9%	29	24	45.3%
Claude	4	54	98.3%	3	55	94.8%	0	53	100%
Gemini	0	58	100%	0	58	100%	0	53	100%

#### 4.1.1 Modified word frequency test

**Figure 2:**
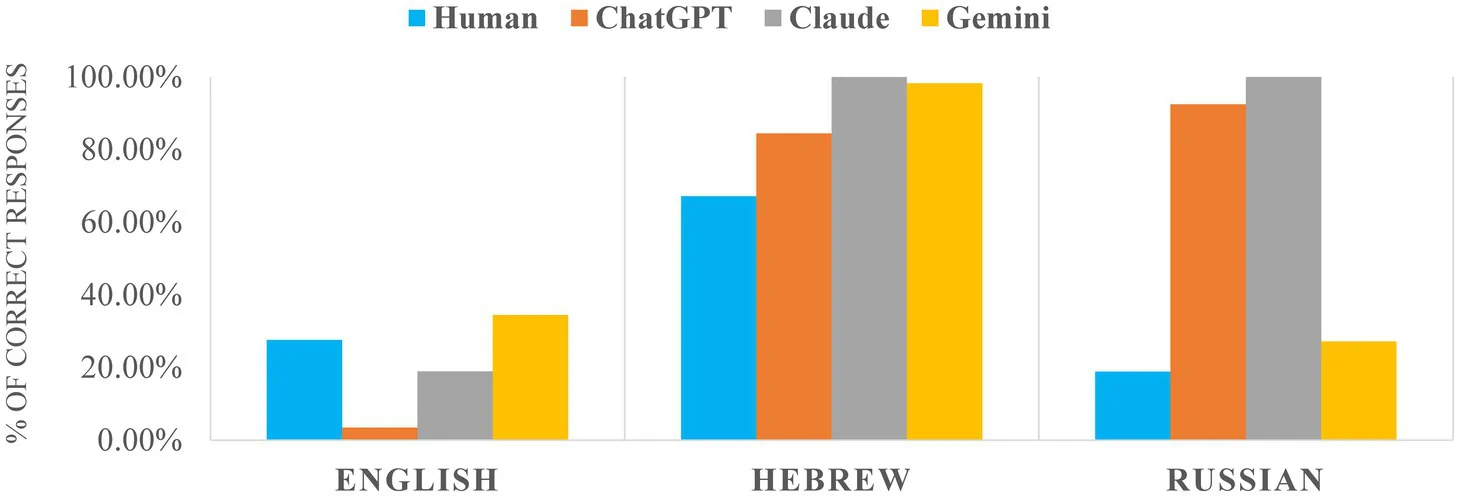
Modified word frequency test language-source interaction plot (%Correct).

For English, the results showed a significant association between the source of response and its correctness, *χ*^2^(3, *N* = 232) = 18.71, *p* < 0.001, with a small-to-medium effect size (Cramér’s V = 0.28). Overall accuracy was low across all sources. ChatGPT showed extremely low performance (3.5%), while Claude (18.9%) and Humans (27.6%) performed at similarly low levels. Gemini achieved the highest accuracy in English (34.5%), though performance levels remained relatively modest. For Hebrew, the association remained highly significant, *χ*^2^(3, *N* = 232) = 36.69, *p* < 0.001 with a medium effect size (Cramér’s V = 0.40). Claude achieved perfect accuracy (100%), followed closely by Gemini (98.3%) and ChatGPT (84.5%), while Humans displayed less accurate performance (67.2%). For Russian, the results again revealed a significant association, *χ*^2^(3, *N* = 212) = 115.94, *p* < 0.001, with a large effect size (Cramér’s V = 0.74). Claude (100%) and ChatGPT (92.5%) showed a near-ceiling performance. In contrast, Gemini’s accuracy was substantially lower (27.2%), though it still exceeded human performance (18.9%) (see Figure 2).

#### 4.1.2 Original word frequency test

**Figure 3:**
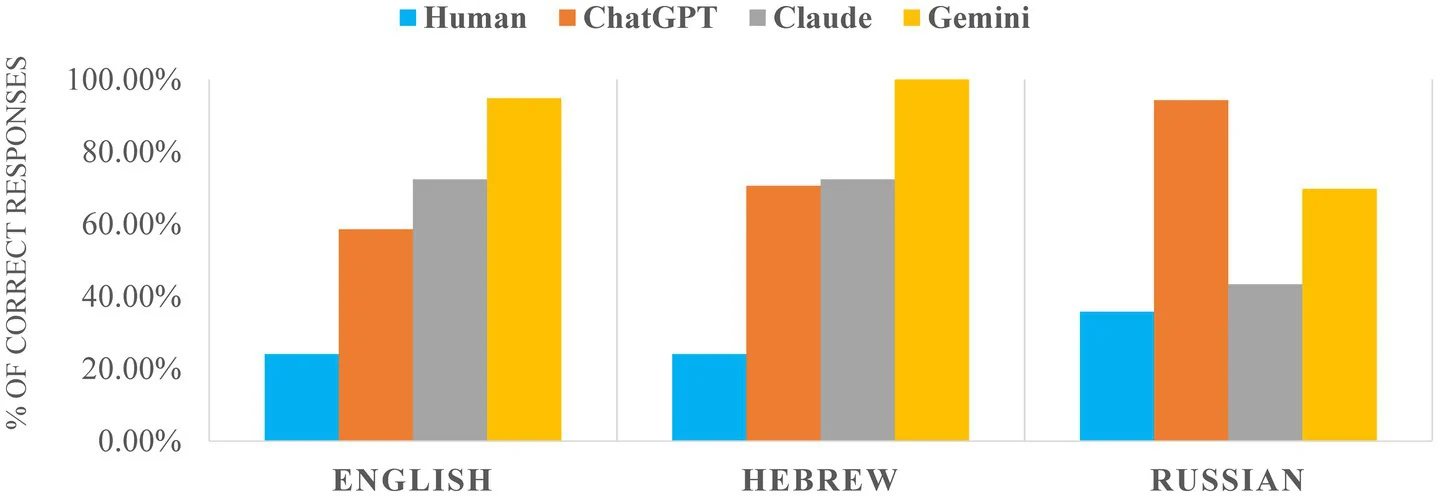
Original word frequency test language-source interaction plot (%Correct).

For English, the association between response source and correctness was highly significant, *χ*^2^(3, *N* = 232) = 65.09, *p* < 0.001 with a large effect size (Cramér’s V = 0.53). Gemini achieved the highest accuracy (94.8%), followed by Claude (72.4%) and ChatGPT (58.6%), whereas Humans performed substantially worse (24.1%). For Hebrew, the results also showed a highly significant association, *χ*^2^(3, *N* = 232) = 77.66, *p* < 0.001 with a large effect size (Cramér’s V = 0.58). Gemini demonstrated perfect accuracy (100%), followed by Claude (72.4%) and ChatGPT (70.6%). Human accuracy remained low (24.1%). For Russian, the association was again significant, *χ*^2^(3, *N* = 212) = 47.42, *p* < 0.001 with a medium-to-large effect size (Cramér’s V = 0.47). ChatGPT (94.3%) outperformed Gemini (69.8%) and Claude (43.4%), while human participants again showed the lowest accuracy (35.8%) (see Figure 3).

#### 4.1.3 Modified Wason selection task

**Figure 4:**
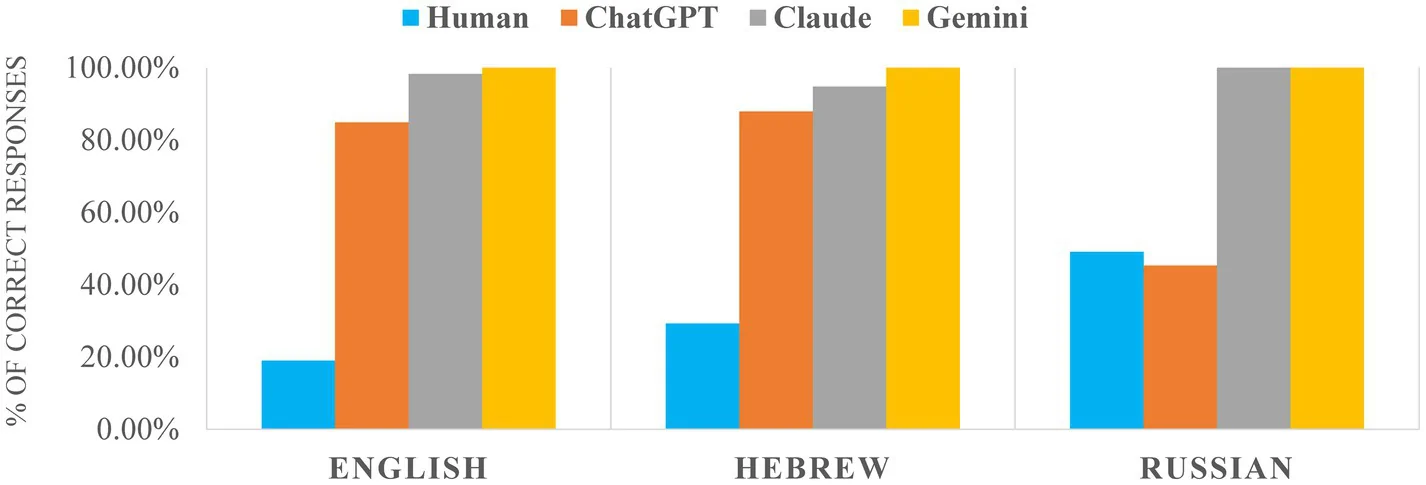
Wason selection task language-source interaction plot (%Correct).

For English, the association between response source and correctness was highly significant, χ^2^(3, *N* = 232) = 126.43, *p* < 0.001, with a large effect (Cramér’s V = 0.74). Gemini (100%) and Claude (98.3%) demonstrated near-perfect logical reasoning, ChatGPT also exhibited high accuracy (84.9%), and Humans scored lowest (19.0%). For Hebrew, the association was again highly significant, χ^2^(3, N = 229) = 109.45, *p* < 0.001, with a large effect size (Cramér’s V = 0.69). Gemini and Claude achieved perfect and near-perfect accuracy (100 and 94.8%, respectively) and ChatGPT again showed highly accurate performance (87.9%). Human performance remained substantially lower (29.3%). For Russian, the results showed a highly significant association, χ^2^(3, N = 212) = 78.39, *p* < 0.001, with a large effect (Cramér’s V = 0.61). Both Claude and Gemini achieved perfect accuracy (100%), while human participants (49.1%) exhibited near-chance performance levels and ChatGPT scored even lower (45.3%) (see Figure 4).

### 4.2 *Post hoc* pairwise comparisons

Next, following the significant Chi-Square test result in all cognitive tasks, a series of *post hoc* pairwise comparisons were conducted to precisely identify which groups differed in their rates of correct (TRUE) responses. To address the risk of multiple-comparison false positives, the Bonferroni correction was applied to all *p*-values.

### 4.2.1 Modified word frequency test

For English, post hoc comparisons indicated that ChatGPT performed significantly worse than human participants (*p* = 0.005) and Gemini (*p* < 0.001). No significant differences were observed between Humans, Gemini and Claude (all *p* = 1.000), nor between ChatGPT and Claude (*p* = 0.111), indicating broadly comparable performance between these pairs after correction. In Hebrew, Claude significantly outperformed both Humans and ChatGPT (*p* < 0.001 and *p* = 0.033, respectively), while Humans and ChatGPT formed significantly indistinguishable group (*p* = 0.305). Gemini performed significantly better than human control group (p < 0.001), but did not differ significantly from Claude or ChatGPT after correction. For Russian, ChatGPT and Claude did not differ significantly from one another (*p* = 1.000) and both significantly outperformed human participants (*p* < 0.001). Both models also significantly outperformed Gemini (*p* < 0.001), while Gemini did not differ significantly from Humans (*p* = 1.000).

#### 4.2.2 Original word frequency test

For English, the results indicated two distinct performance tiers: ChatGPT and Claude performance did not differ significantly from one another (*p* = 1.000), and Human and Gemini formed significantly indistinguishable pair as well (*p* = 1.000). The comparison between the two tiers was highly significant (*p* < 0.001). For Hebrew, ChatGPT, Claude and Gemini each differed significantly from Human responses (all *p* < 0.001). ChatGPT and Claude did not differ significantly from one another (*p* = 1.000). However, Gemini performed significantly better than both ChatGPT (*p* < 0.001) and Claude (*p* < 0.001). For Russian, ChatGPT’s near perfect accuracy exceeded that of Humans (*p* < 0.001), Claude (*p* < 0.001), and Gemini (*p* < 0.001). Claude’s performance did not differ significantly from Humans (*p* = 1.000), whereas Gemini performed significantly better than Humans (*p* = 0.006), but did not differ significantly from Claude after correction (*p* = 0.065).

#### 4.2.3 Modified Wason selection task

For English, the results revealed a clear performance hierarchy. Humans performed significantly worse than ChatGPT (*p* < 0.001), Claude (*p* < 0.001), and Gemini (*p* < 0.001). ChatGPT did not differ significantly from Claude (*p* = 1.000) but performed significantly worse than Gemini (*p* = 0.033). No significant difference was observed between Claude and Gemini (*p* = 0.761), indicating comparable near-ceiling performance between these two models. For Hebrew, Human performance was significantly lower than all three models (all *p* < 0.001). ChatGPT did not differ significantly from Claude (*p* = 1.000) or from Gemini (*p* = 0.116). No significant difference was observed between Claude and Gemini (*p* = 1.000), indicating comparable high performance among the three models. For Russian, no significant differences were found between human participants and ChatGPT (*p* = 1.000), indicating comparable near-chance performance. In contrast, both Claude and Gemini performed significantly better than Humans (both *p* < 0.001) and ChatGPT (both *p* < 0.001), while no significant difference was found between Claude and Gemini since both achieved perfect accuracy (*p* = 1.000).

### 4.3 Logistic regression analysis

Finally, to provide a more nuanced understanding of differences in performance and to quantify the effect of each LLM, a binary logistic regression model was developed for each cognitive task. The model predicted the log-odds of providing a correct (TRUE) response using the source of the response (Human, ChatGPT, Claude, Gemini) as the sole predictor variable. To create a meaningful comparison, the performance of Human participants served as the reference category, namely, each LLM’s performance was evaluated relative to the human baseline. Since some language–model combinations demonstrated imbalanced outcome distributions, including near-perfect and extremely low accuracy rates, Firth penalized logistic regression was applied to reduce potential separation effects and stabilize coefficient estimation.

#### 4.3.1 Modified word frequency test

For English, the human baseline was significant (*B* = −0.965, *p* = 0.001, OR = 0.38), indicating a higher likelihood of incorrect responses. ChatGPT performed significantly worse than humans (*B* = −2.367, *p* = 0.002, OR = 0.09). Claude and Gemini did not differ significantly from humans (*p* = 0.274 and *p* = 0.423, respectively). For Hebrew, the human baseline was significant (*B* = 0.719, *p* = 0.010, OR = 2.05), showing higher odds of correct responses. ChatGPT performed significantly better than humans (*B* = 0.976, *p* = 0.0332, OR = 2.652). Claude exhibited complete separation due to perfect accuracy (*p* = 0.989), whereas Gemini showed significantly higher odds of correct responses relative to Humans (*B* = 3.324, *p* = 0.0015, OR = 27.77). Firth penalized logistic regression was therefore applied to address separation effects. The penalized model yielded similar findings, confirming significantly greater odds of correct responses for ChatGPT (B = 0.945, *p* = 0.031, OR = 2.57), Claude (*B* = 4.056, *p* < 0.001, OR = 57.76), and Gemini (B = 2.940, *p* < 0.001, OR = 18.92) relative to humans.

For Russian, the human baseline was significant (*B* = −1.459, *p* < 0.001, OR = 0.23), indicating low accuracy among participants. ChatGPT performed significantly better than humans (*B* = 3.964, *p* < 0.001, OR = 52.68). Gemini did not differ significantly (*p* = 0.179), while Claude’s perfect accuracy again resulted in an unstable coefficient (*p* = 0.989) due to complete separation. In the penalized model, the human baseline remained significant (*B* = −1.421, *p* < 0.001, OR = 0.24). ChatGPT and Claude showed significantly greater odds of correct responses relative to Humans (*B* = 3.819, *p* < 0.001, OR = 45.57) and (*B* = 6.094, *p* < 0.001, OR = 443.29), respectively, whereas Gemini did not significantly differ from the human baseline (*p* = 0.181, OR = 1.82).

#### 4.3.2 Original word frequency test

For English, the human baseline was significant (*B* = −1.145, *p* < 0.001, OR = 0.32), indicating that participants were more likely to respond incorrectly than correctly. ChatGPT (*B* = 1.493, *p* < 0.001, OR = 4.45), Claude (*B* = 2.110, *p* < 0.001, OR = 8.25), and Gemini (*B* = 4.054, *p* < 0.001, OR = 57.62) performed significantly better than humans, showing substantially higher odds of correct responses. For Hebrew, the human baseline was again significant (*B* = −1.145, *p* < 0.001, OR = 0.32), reflecting lower odds of correct responses among participants. ChatGPT (*B* = 2.026, *p* < 0.001, OR = 7.58) and Claude (*B* = 2.110, *p* < 0.001, OR = 8.25) significantly outperformed humans. Gemini exhibited complete separation due to perfect accuracy, resulting in an unstable coefficient (*p* = 0.981). Firth penalized logistic regression was therefore applied to address separation effects, confirming significantly greater odds of correct responses for ChatGPT (*B* = 1.985, *p* < 0.001, OR = 7.28), Claude (*B* = 2.067, *p* < 0.001, OR = 7.90), and Gemini (*B* = 5.866, *p* < 0.001, OR = 352.93) relative to humans. For Russian, the human baseline was marginally significant (*B* = −0.582, *p* = 0.042, OR = 0.56), indicating slightly lower odds of correct responses. ChatGPT (*B* = 3.395, *p* < 0.001, OR = 29.82) and Gemini (*B* = 1.420, *p* < 0.001, OR = 4.14) performed significantly better than Humans. Claude did not differ significantly from the Human baseline (*p* = 0.428).

#### 4.3.3 Modified Wason selection task

For English, the human baseline was significant (*B* = −1.452, *p* < 0.001, OR = 0.23), indicating a higher likelihood of incorrect responses. All three models significantly outperformed humans. ChatGPT showed higher odds of correct responses (*B* = 3.147, p < 0.001, OR = 23.26), while Claude demonstrated an even larger advantage (*B* = 4.055, *p* < 0.001, OR = 57.68), and Gemini achieved perfect accuracy, resulting in complete separation and an unstable coefficient (*p* = 0.988). Firth penalized logistic regression was therefore applied and produced similar findings − significantly greater odds of correct responses for ChatGPT (*B* = 3.069, *p* < 0.001, OR = 21.52), Claude (*B* = 3.913, *p* < 0.001, OR = 50.02), and Gemini (*B* = 6.181, *p* < 0.001, OR = 483.26) relative to humans. For Hebrew, the human baseline was significant (*B* = −0.880, *p* = 0.002, OR = 0.41). Both ChatGPT (*B* = 2.866, *p* < 0.001, OR = 17.57) and Claude (*B* = 3.789, *p* < 0.001, OR = 44.22) significantly outperformed humans. Gemini once more achieved perfect accuracy, yielding an unstable coefficient due to complete separation (*p* = 0.988). Firth penalized logistic regression again produced comparable findings, with ChatGPT (*B* = 2.790, *p* < 0.001, OR = 16.28), Claude (*B* = 3.627, *p* < 0.001, OR = 37.60), and Gemini (*B* = 5.626, *p* < 0.001, OR = 277.46) maintaining significantly greater odds of correct responses relative to humans. For Russian, the human baseline was not significant (*B* = −0.038, *p* = 0.891, OR = 0.96), indicating no systematic bias toward correct or incorrect responses. ChatGPT did not differ significantly from human participants (*B* = −0.228, *p* = 0.559, OR = 0.79), reflecting comparable near-chance performance, while both Claude and Gemini achieved perfect accuracy, resulting in complete separation and unstable coefficients (both *p* = 0.989). Firth penalized logistic regression confirmed that Claude and Gemini had significantly greater odds of correct responses relative to Humans (both *B* = 4.710, *p* < 0.001, OR = 111.04). ChatGPT remained non-significant in the penalized model (*B* = −0.149, *p* = 0.700, OR = 0.86).

### 4.4 LLMs’ cross-linguistic performance

As an initial step, a language-specific, model-level performance analysis was conducted by aggregating responses across all three cognitive tasks (Modified and Original Word Frequency tests and the Wason Selection task). The second part of the analysis focused on the breakdown of each model’s performance across languages and tasks in an attempt to isolate the nuances underlying the models’ possible susceptibility to different types of cognitive biases (see Table 3).

**Table 3:** Contingency table of overall response correctness by LLM and language.

Source	English			Hebrew			Russian		
	Incorrect	Correct	%Correct	Incorrect	Correct	%Correct	Incorrect	Correct	%Correct
ChatGPT	89	85	48.3%	33	141	81.0%	36	123	77.4%
Claude	67	107	61.5%	19	155	89.1%	30	129	81.1%
Gemini	41	133	76.4%	1	173	99.4%	53	106	66.7%

**Figure 5:**
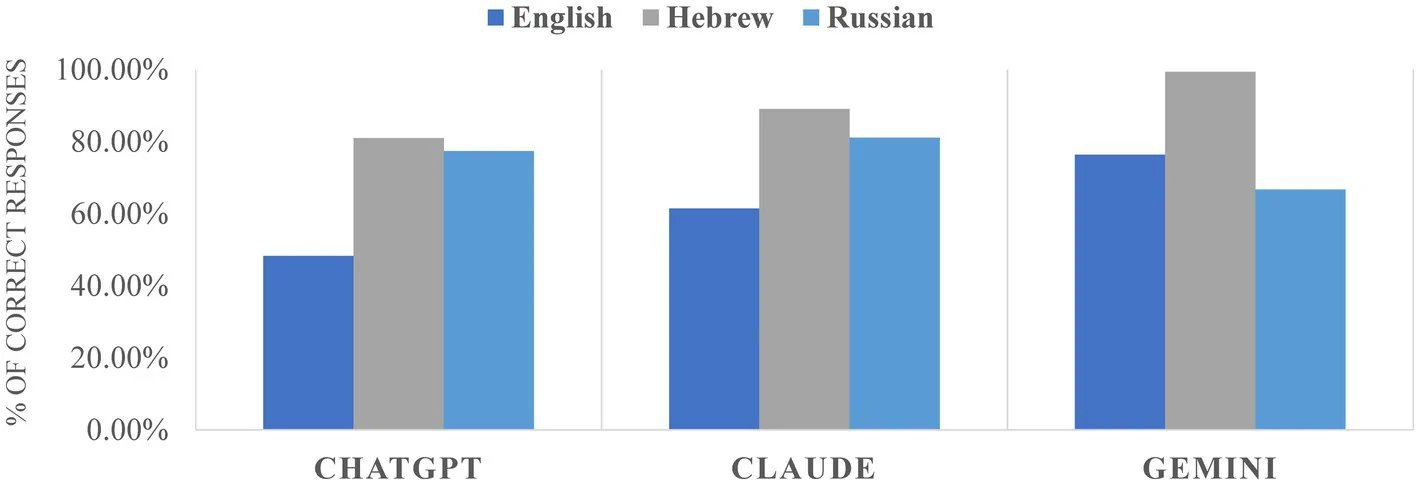
Model-language interaction plot (%Correct).

Pearson’s Chi-Square tests for independence were conducted for each model to examine the relationship between the source language (English, Hebrew, Russian) and response correctness. For all three models, the results showed a highly significant language effect: For ChatGPT—*χ*^2^(2, *N* = 510) = 49.85, *p* < 0.001, with a medium effect size (Cramér’s V = 0.314). Models’ accuracy was highest in Hebrew (81.0%), followed by comparable performance in Russian (77.4%), and moderate accuracy in English (48.3%), revealing cross-linguistic variation. For Claude—*χ*^2^(2, *N* = 477) = 39.64, *p* < 0.001, with a small-to-medium effect size (Cramér’s V = 0.28). The model achieved high accuracy in Hebrew (89.1%), followed by Russian (81.1%), while English performance was comparatively moderate (61.5%), forming two distinct tiers of proficiency. For Gemini—*χ*^2^(2, *N* = 477) = 62.60, *p* < 0.001, with a medium effect size (Cramér’s V = 0.35). The model achieved a near-ceiling accuracy in Hebrew (99.4%), while showing less accurate performance in both English (76.4%), and Russian (66.7%) (see Figure 5).

Following the significant Chi-Square test results, the subsequent phase focused on a *post hoc* pairwise comparison, which identified the following differences in model performance across specific languages:

For ChatGPT, the analysis partitioned the groups into two distinct performance tiers. Model’s performance in English was significantly less accurate than its performance in Hebrew (*p* < 0.001) and Russian (*p* < 0.001), whereas performances in Russian and in Hebrew did not differ significantly (*p* = 1.000). For Claude, the analysis confirmed a similar performance hierarchy - the model’s accuracy in English was significantly lower than in Russian (*p* < 0.001) and in Hebrew (*p* < 0.001), while accuracy in Russian and Hebrew did not differ significantly (*p* = 0.176). For Gemini, the analysis identified that in Hebrew the model significantly outperformed Russian (*p* < 0.001) and English (*p* < 0.001), whereas the performances in English and Russian were not found to be significantly different (*p* = 0.190).

#### 4.4.1 LLMs’ cross-linguistic performance across specific tasks

Pearson’s Chi-Square test for independence was used to examine the relationship between the source language (English, Hebrew, and Russian) and response correctness for each model and each cognitive task.

##### 4.4.1.1 Modified word frequency test

**Figure 6:**
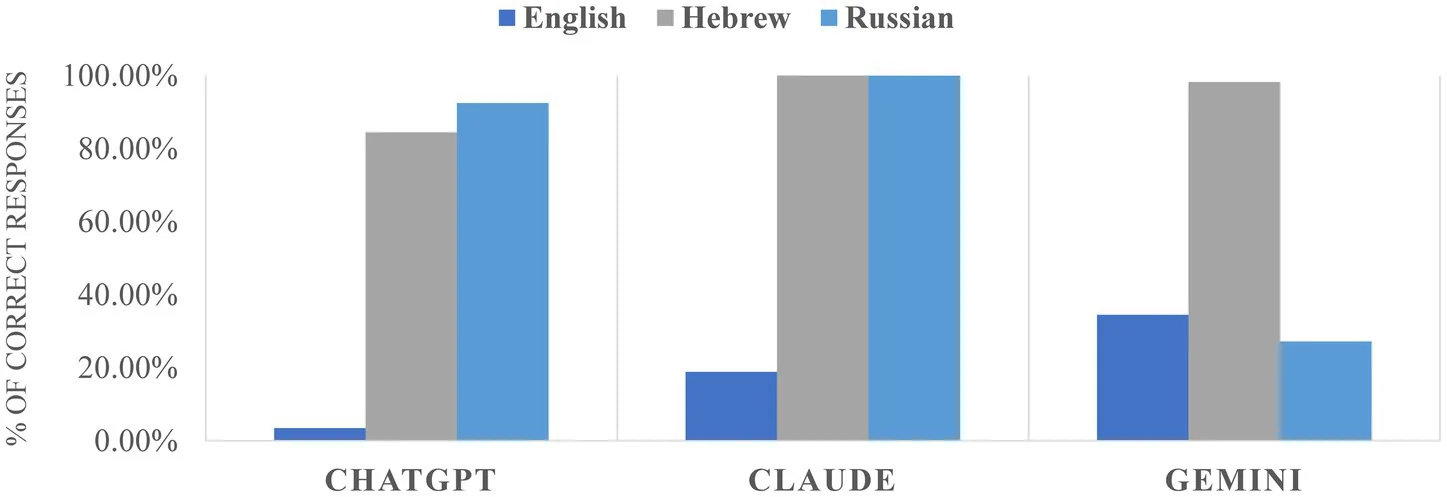
Modified word frequency test model-language interaction plot (%Correct).

The results revealed highly significant language effects for all models, exhibiting a consistent pattern of high and low performance tiers across languages: ChatGPT—*χ*^2^(2, *N* = 169) = 114.23, *p* < 0.001, with a large effect size (Cramér’s V = 0.82). The model’s accuracy was highest in Russian (92.5%), followed by very accurate performance in Hebrew (84.5%). In contrast, model’s accuracy in English was very low (3.5%). Claude—*χ*^2^(2, *N* = 169) = 124.60, *p* < 0.001, with a large effect size (Cramér’s V = 0.86). The model achieved perfect accuracy in Russian (100%) and Hebrew (100%), while displaying substantially lower accuracy in English (18.9%). Gemini—*χ*^2^(2, *N* = 169) = 66.94, *p* < 0.001, with a large effect size (Cramér’s V = 0.629). The model achieved its highest accuracy in Hebrew (98.3%), while performance was substantially lower and comparable in English (34.5%) and Russian (30.2%) (see Figure 6).

##### 4.4.1.2 Original word frequency test

**Figure 7:**
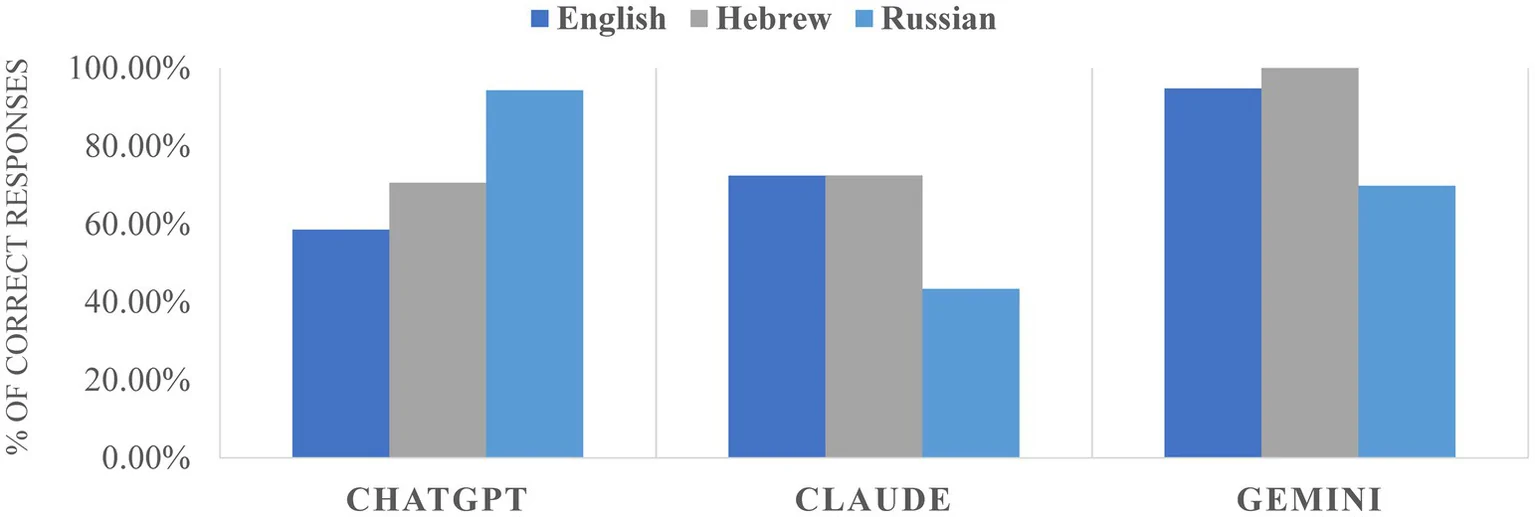
Original word frequency test model-language interaction plot (%Correct).

All models showed highly significant language effects: ChatGPT—*χ*^2^(2, *N* = 169) = 18.84, *p* < 0.001, with a medium effect size (Cramér’s V = 0.334). Its accuracy was almost perfect in Russian (94.3.7%), high in Hebrew (70.6%), and moderate in English (58.6%), suggesting a performance gradient across languages. Claude—*χ*^2^(2, *N* = 169) = 13.19, *p* = 0.001, with a small-to-medium effect size (Cramér’s V = 0.279). The model displayed equal accuracy levels in both in English and Hebrew (72.4%) and much lower accuracy in Russian (43.4%). Gemini—*χ*^2^(2, *N* = 169) = 28.55, *p* < 0.001, with a medium effect size (Cramér’s V = 0.41). Accuracy was highest in Hebrew (100%), followed by English (94.8%), and was substantially lower in Russian (69.8%) (see Figure 7).

##### 4.4.1.3 Modified Wason selection task

**Figure 8:**
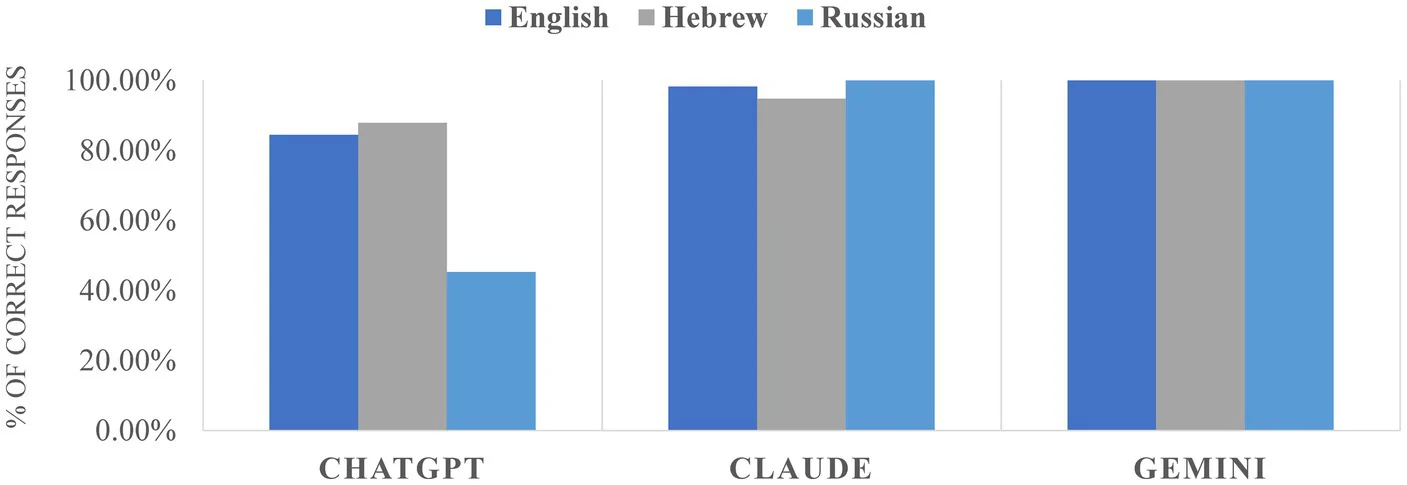
Modified Wason selection task model-language interaction plot (%Correct).

For ChatGPT the results revealed a highly significant association between language and response correctness—*χ*^2^(2, *N* = 169) = 31.36, *p* < 0.001 with a medium effect size (Cramér’s V = 0.431). The models’ accuracy was highest in Hebrew (87.9%) and English (84.5%), while performance in Russian was substantially lower (45.3%), indicating a pronounced language-dependent variation. No significant language effects were observed for Claude or Gemini due to their near perfect performance across all languages (see Figure 8).

Following significant Chi-Square results, *post-hoc* pairwise comparisons were conducted to identify which LLMs differed across source languages. Bonferroni corrections were applied for multiple comparisons.

For the Modified Word Frequency Test all models showed a strong language-dependent pattern. ChatGPT achieved significantly higher accuracy in Hebrew and Russian than in English (*p* < 0.001), with no difference between Hebrew and Russian (*p* = 1.000). Claude showed significantly lower performance in English than in both Russian and Hebrew (both *p* = 0.011), while Russian and Hebrew did not differ significantly (*p* = 1.000). Gemini displayed significantly higher accuracy in Hebrew than in English and Russian (both *p* < 0.001), whereas English and Russian did not differ significantly (*p* = 1.000).

In the Original Word Frequency Test, *post-hoc* comparisons showed that ChatGPT performed significantly worse in English and Hebrew than in Russian (both *p* < 0.001), with a small but statistically significant difference between English and Hebrew (*p* = 0.001). Claude showed significantly lower performance in Russian than in both English and Hebrew (both *p* = 0.011), whereas no significant difference was found between English and Hebrew (*p* = 1.000). Gemini demonstrated a significantly lower performance in Russian than in both English (*p* = 0.004) and Hebrew (*p* < 0.001). No significant difference was observed between English and Hebrew performance (*p* = 0.726).

As for the Modified Wason Selection Task, ChatGPT in English and Hebrew significantly overperformed Russian (both *p* < 0.001), while no significant difference was observed between model’s performance in English and Hebrew (*p* < 0.001). Both Claude and Gemini did not display significant pairwise differences in language, suggesting uniform performance across all three languages for these models.

## 5 Discussion

This study investigated whether cognitive biases, such as availability heuristic and confirmation bias, are reflected in the outputs of Large Language Models (ChatGPT, Claude, Gemini) and whether prompt language affects the expression of these biases when identical cognitive tasks are administered in different languages (English, Hebrew, Russian).

The research findings revealed consistent, though non-uniform, evidence of availability heuristics in the models’ responses to word-frequency tasks, corroborating and extending prior research (Berberette et al., 2024; Kliegr et al., 2021), and confirming one of the study’s hypotheses. In contrast, the models demonstrated markedly efficient performance in confirmation bias task: confirmation-like behavior was observed only in a single experimental condition (ChatGPT performance in Russian), while in all remaining conditions LLMs achieved significantly higher accuracy than human control group, suggesting a more limited and context-based effect than previously reported (Chen et al., 2025; Macmillan-Scott and Musolesi, 2024; Schmidgall et al., 2024). These results reveal a consistent pattern that implies a presence of a cognitive gap: LLMs tend to behave like high-performing rule-followers in deductive tasks, such as the Wason Selection Task, while demonstrating systematic language- and model-dependent differences in reasoning-based tasks, such as both Word Frequency Tasks. Crucially, the significant language effects that emerged across all three experimental tools—the Modified and the Original Word Frequency Tests and the Wason Selection Task, with effect sizes ranging from medium to large, could indicate that the degree of bias susceptibility might be a result of interactions between model-specific characteristics and linguistic context rather than a universal LLM tendency. In practical terms, this means that model’s performance observed in English in a specific cognitive task is a poor predictor of the same model’s performance in Hebrew or in Russian, and vice versa (see Table 4).

**Table 4:** Heatmap of overall results.

Language	Modified word frequency test				Original word frequency test				Wason selection task			
	Human	GPT	Claude	Gemini	Human	GPT	Claude	Gemini	Human	GPT	Claude	Gemini
English	28%	4%	19%	35%	24%	59%	72%	95%	19%	85%	98%	100%
Hebrew	67%	85%	100%	98%	24%	71%	72%	100%	29%	88%	95%	100%
Russian	19%	93%	100%	27%	36%	94%	43%	70%	49%	45%	100%	100%

The heatmap that displays gradation of color from green for the highest percentage of correct asnwers to red for the lowest.

### 5.1 LLM performance in word frequency tasks

While this study found evidence of availability-like patterns in LLMs’ outputs, its effect varied across models and languages, aligning, in part, with prior findings, while clarifying specific conditions—of model, language, and task—under which this bias might appear or diminish.

The analysis of LLMs’ performances in the Modified Word Frequency Test in English showed that Claude and Gemini produced responses statistically indistinguishable from the human baseline – i.e., presented “human-like” availability effects, as operationalized in the present study (Macmillan-Scott and Musolesi, 2024). In contrast, ChatGPT’s struggled significantly compared to humans, suggesting that its errors were not just reflections of heuristic shortcuts; instead, the difficulty seemed tied to the specific combination of task and language. For Hebrew task, the pattern was somewhat different—Claude and Gemini no longer mirrored human performance but instead significantly outperformed both humans and ChatGPT, who achieved similar accuracy. Russian results further indicated model-language dependence of availability manifestation. In this condition, Gemini’s responses were not significantly different from the human control group, indicating comparable heuristic behavior, while Claude and ChatGPT significantly outperformed both groups.

The second research tool, i.e., Original Word Frequency Test, also produced varied results. In English the test revealed two distinct performance tiers with Claude and ChatGPT forming a high-performance group, while Gemini and human control group exhibited statistically indistinguishable performance levels, indicating comparable manifestation of availability heuristics in their responses. In Hebrew, all three LLMs significantly outperformed human control group and in Russian the performance was again split into two tiers: Claude performed at human level, whereas both ChatGPT and Gemini achieved significantly higher accuracy.

The variability of these results offers several important insights into the way that contemporary LLMs process information and generate responses when presented with cognitive tasks. The most significant finding is that availability heuristic does not appear to be a context-invariant flaw but instead emerges from interactions between types of LLMs’ architecture and the specific properties of prompt languages. Since all models were explicitly instructed to rely on general reasoning rather than data retrieval, the observed results should not be simply attributed to differences in training data or web representation. Instead, these patterns likely reflect an interaction between the model’s learned priors and the linguistic structure of the input (morphology, lexical dispersion, etc.). It’s possible that specific language traits shift the model’s attention mechanism—if certain cues become more or less obvious, it changes the way the model relies on simple frequency shortcuts instead of deeper reasoning. The overall strong and consistent performance in Hebrew for both Word Selection tasks is particularly telling, suggesting that certain language-specific structural features might reduce availability-style shortcuts that were activated in English or in Russian.

### 5.2 LLM performance in Wason selection task

The analysis of the Wason Selection Task did not reveal uniform evidence of biased responses in the outputs of the tested LLMs. In English and Hebrew, the results showed a clear separation between human participants and model performance—humans consistently underperformed across both languages, while models approached the task in a stable, rule-consistent manner. In contrast, in Russian, while Claude and Gemini achieved perfect accuracy, ChatGPT did not differ significantly from the human control group. This condition-dependent result further supports the interpretation that observed differences in LLMs performance reflect models’ architectural and language-handling properties rather than systematic bias.

ChatGPT-4o demonstrated markedly improved accuracy in the Modified Wason Selection Task when administered in English and Hebrew, a stark departure from the consistently poor performance reported for GPT-3.5 and GPT-4 in comparable logical reasoning tasks (Chen et al., 2025). This cross-generational improvement likely reflects refinements in instruction-tuning protocols and the incorporation of bias-mitigation strategies during training, which together may enhance the model’s capacity for rule-based deductive reasoning. Critically, however, this improvement was not observed uniformly across all language conditions. ChatGPT-4o’s performance on the identical task in Russian remained at near-chance levels, statistically indistinguishable from the human control group. This language-specific performance gap undermines any interpretation of uniform cognitive advancement and instead reinforces the central finding of this study: reasoning capability in LLMs is fundamentally conditioned by linguistic context. Model generation, while potentially indicative of architectural sophistication, does not guarantee consistent reasoning performance across languages.

The almost perfect performance of LLMs in Wason Selection tasks stands in contrast to the revealed consistent availability-driven patterns that were present across models and languages in Word Selection tasks. This suggests that cognitive biases in LLMs are selective rather than global: models may avoid confirmation tendencies in logical tasks (e.g., if-p-then-q), and yet exhibit availability-style responses in heuristic settings, with their expression shaped by the interplay of model, language, and task.

The task’s deontic framing warrants consideration as a potential confounding factor in interpreting the observed performance patterns. Deontic versions of the Wason Selection Task, those involving social rules or obligations rather than abstract logical relations, consistently elicit improved accuracy in both human participants and language models, a robust effect termed ‘deontic facilitation’ (Abe et al., 2026). The mechanism underlying this effect likely involves the activation of domain-specific reasoning schemas related to social contract verification, which are more readily accessible than pure logical deduction. In the present study, the use of a deontic framing (airline passenger verification) may have contributed to the exceptionally high accuracy rates observed for Claude and Gemini in English and Hebrew. However, this explanation alone cannot account for the full pattern of results. If deontic facilitation operated uniformly, we would expect elevated performance across all models and languages. Instead, ChatGPT’s performance in Russian remained at near-chance levels, statistically indistinguishable from the human baseline. This divergence suggests that deontic facilitation in LLMs is not a fixed property of the task but rather emerges from a three-way interaction among task framing, model architecture, and linguistic context. Future research should systematically manipulate task framing (deontic vs. abstract) across multiple languages to isolate the relative contributions of these factors to reasoning performance.

### 5.3 LLMs’ cross-linguistic performance patterns

The results across all three languages show that LLMs do not maintain a single, steady level of intelligence; instead, their performance shifts significantly depending on whether they are prompted in English, Hebrew, or Russian. This corroborates the research hypothesis that language is far more than just a translation layer—it influences how a model thinks through a problem. Even with explicit instructions to ignore outside data, the differences in language structure seem to “pull” the models toward different reasoning paths. This suggests that the way LLMs approach a task might be tied to the linguistic framework it is operating in at that moment. The finding complements, rather than overturns, previous assumptions that attributed the most significant source of bias, regardless of its type, to the training dataset and its quality (Mondal and Lipizzi, 2024) and suggests that a more nuanced understanding of AI performance is required. Namely, one that looks beyond training data quality and accounts for the prompt language as an active factor that can shape LLMs’ reasoning process.

While research findings propose the language-dependent processing interpretation, they also imply an absence of a single best-performing language. Contrary to the common presumption that English should dominate by virtue of its historically central role in computational linguistics (Maksymenko and Turuta, 2025) it was often the weakest performer, most notably for all three models (especially ChatGPT) in the Modified Frequency Test and for ChatGPT and Claude in overall performance evaluation. On the contrary, all models yielded the highest accuracy in Hebrew, while their performance in Russian proved to be very variable and task-dependent—best under some conditions, worst under another (most notably in Modified Wason Selection Task). These findings suggest that language proficiency does not directly translate into superior reasoning in cognitive tasks. Instead, model-internal logic seems to interact with language-specific structure in complex ways, producing performance variability across languages.

Further analysis showed that each model’s performance also depended on the interaction between the language and the type of cognitive task. The hierarchy was unstable across tasks, with Claude excelling in Russian overall but scoring poorly on the Original Word Frequency Test compared to ChatGPT and Gemini. This implies that specific features of a language’s syntax or semantics may either facilitate or obstruct the model’s ability to solve a particular type of logical test. Perfect scores for Claude and Gemini on the Wason Selection task versus confirmation-consistent response patterns in ChatGPT in Russian further support this interpretation.

Collectively, the findings of this research demonstrate that machine intelligence is not a generic resource. When tested under identical tasks and constraints, Gemini, Claude, and ChatGPT exhibited distinct response profiles. Each model’s internal design appeared to interact with prompt language in unique ways, making architectural differences an essential consideration when evaluating reasoning capabilities. Linguistic context also appears to shape LLM reasoning rather than simply serve as a neutral medium. Language seems to influence model accuracy across cognitive tasks, creating shifting and unpredictable performance hierarchies. The absence of one superior language challenges assumptions about linguistic dominance in LLM reasoning, suggesting that model architecture, task characteristics, and language-specific features jointly influence behavior. From this perspective, cognitive biases and reasoning errors do not just come from training data—they may also emerge in real-time as models interact with the specific linguistic structure of each prompt.

### 5.4 Research implications

First, the findings of this research suggest multiple avenues for bias mitigation. At the prompt level, explicit instructions targeting specific biases may reduce bias expression. At the model level, interventions might include balancing training data across languages, calibrating output parameters, and incorporating bias-specific evaluation steps in reasoning chains. Importantly, cross-linguistic transfer of bias resistance should not be assumed; models that appear immune to specific biases in one language may exhibit such biases when operating in different linguistic contexts.

Second, the systematic cross-linguistic variation observed across all tasks challenges the assumption of language-invariant reasoning in Large Language Models, revealing that linguistic context might shape how these models approach reasoning tasks. This implies that users cannot assume consistent performance of Large Language Models when switching between languages and should validate critical outputs by testing the same queries across multiple languages or by seeking verification through alternative reasoning approaches. From a systems and managerial perspective, these findings point to the need for a shift in AI implementation strategies. Organizations should consider extending validation and testing frameworks beyond single-language benchmarks to establish comprehensive multilingual validation processes before deployment. This implies incorporating natural language as a formal variable in model evaluation, as well as designing control mechanisms that account for systematic performance variability across linguistic contexts. In other words, language should be treated as a core design parameter in AI systems, with safeguards and monitoring protocols tailored to each linguistic context.

Third, this research adds an important dimension to the discussion on the role of Large Language Models as experts that influence decision-making processes. The lack of stability in models’ performance across tasks and languages demonstrates that LLMs cannot serve as consistently reliable sources of judgment. As this research suggests, models’ outputs may exhibit cognitive bias-consistent response patterns, such as availability heuristics, or deviate significantly from correct reasoning, leading to unpredictable outputs. The observed variability is particularly problematic given evidence that users, especially those with lower confidence, may be disproportionately influenced by AI recommendations (Gomez et al., 2024) and prone to overreliance in critical contexts (Ma et al., 2025). Such overreliance on inconsistent outputs may compromise decision quality, leading users to adopt the model’s biases or errors as their own. Therefore, responsible LLM use requires individuals to develop essential skills of evaluation, approaching LLMs’ outputs as potentially helpful but fallible tools rather than definitive expert guidance.

### 5.5 Limitations and future directions

While this study advances understanding of cross-linguistic bias patterns in LLMs, several limitations constrain the scope and generalizability of its findings. The use of a single prompt per language in each task limits measurement reliability and complicates the interpretation of cross-linguistic effects. Although task clarity and relevance were assessed through expert validation and inter-rater reliability, the single-item design constrains construct validity. Specifically, internal consistency cannot be estimated, and item-specific variation cannot be separated from broader bias-related effects. Additionally, relying on one prompt per language makes it difficult to determine whether observed cross-linguistic differences reflect translation artifacts, prompt-specific wording effects, or genuine language-related differences. Future work should include multiple items and prompt variants per condition, with item and prompt modeled as random effects, building on existing methodology (Lampinen et al., 2024). The near-perfect performance of several models in logical tasks suggests that task complexity may have been insufficient to reveal meaningful reasoning variation across more advanced models. This limitation is especially relevant to the Wason Selection Task, where ceiling effects reduced discrimination between advanced models. Future research should explore more difficult reasoning tasks, such as multi-step or nested conditionals, to reveal performance nuances that simpler tasks may obscure. Although modest (53 Russian vs. 58 English and Hebrew participants), sample size imbalances may have slightly reduced statistical power for cross-linguistic comparisons. Future studies should ensure balanced sample sizes across all language conditions.

In contrast to previous research (Castello et al., 2024; Chen et al., 2024; Lampinen et al., 2024; Macmillan-Scott and Musolesi, 2024), recent evidence indicates that LLM response patterns on the Wason Selection Task might be attributed to matching bias, a tendency to select cards that lexically match the terms mentioned in the rule, rather than to confirmation bias (Abe et al., 2026). The Wason Selection Task version employed in the current experiment uses a single conditional rule in if-p-then-q format, under which confirmation bias and matching bias result in identical incorrect predictions: choosing the cards that match the rule, rather than the cards needed to test whether the rule is false. The two mechanisms are therefore indistinguishable within this design. Since our findings suggest that LLM performance is not language-invariant, future research should test polarity-varied Wason stimuli methodology (Abe et al., 2026) across multilingual settings. Such work could clarify whether the matching bias interpretation is English-specific or whether it displays general cross-linguistic patterns. This research direction would also address the single-item task limitation by introducing multiple variations of the study instrument.

Although all models were specifically instructed to treat each task as unfamiliar and to rely on general reasoning rather than known benchmark solutions, there is no direct way to verify that this instruction was fully followed. Since both the Wason Selection Task and the Original Word Frequency Test are well-established cognitive instruments, some models may have recognized aspects of the task structure from their training data. However, the substantial variation observed across languages within the same model and task suggests that responses were not driven solely by retrieval of fixed benchmark answers. For example, in the Original Word Frequency Test, ChatGPT improved from 58.6% in English to 94.3% in Russian, while Claude’s performance was similarly accurate (72.4%) in both English and Hebrew, dropping to 43.4% in Russian. Nevertheless, prior task recognition cannot be ruled out entirely. Future research should address this limitation by using newly constructed task variants or by adding a preliminary check in which models are asked whether the task resembles any known reasoning problem before providing their answer.

The study’s focus on three languages and three cognitive tasks limits the breadth of generalization. Broader linguistic coverage, especially inclusion of typologically diverse and low-resource languages, could reveal deeper syntactic, morphological, or semantic drivers of bias. Similarly, tasks validated across more varied cultural contexts would help separate universal cognitive mechanisms from culture-specific reasoning patterns embedded in training data. The human control group was recruited through convenience sampling and is not representative of the general population. The sample was highly educated, included a substantial proportion of participants from technology- and computing-related fields, and nearly half reported daily LLM use. As a result, comparisons between LLMs and human participants should be interpreted relative to this specific group rather than as a broad claim about general human cognition. In this study, “human-like” performance refers only to statistical alignment with the current sample’s response patterns. Our findings reflect a technological snapshot: LLMs evolve rapidly, and results may not hold across future model iterations. Continuous updates to architecture and training protocols make it difficult to generalize beyond the technological moment captured here.

LLMs were queried via their respective official APIs rather than chat interfaces. This distinction is important because consumer applications may include additional system prompts, safety layers, routing mechanisms, and session-management features that are not necessarily present in direct API calls. The present results therefore characterize model behavior under controlled API-based conditions and are not directly generalized to consumer interfaces. The experiment design, in which the number of LLM sessions corresponded to the number of human participants, was used to allow statistical comparability across response sources. However, the independence assumption differs for humans and models. Human participants represent a heterogeneous sample, varying in background knowledge, experience, and reasoning strategies. By contrast, repeated LLM sessions in this research are stochastic outputs from the same model; hence differences between them reflect randomness in generation, not independent individuals. This lack of independence is a recognized limitation in the field of machine psychology. One possible solution could be creating “silicon samples” by conditioning the models on backstories from real human participants (Argyle et al., 2023).

Building on these limitations, several promising research directions emerge. Future studies should employ multiple prompt variants across diverse language families, include bilingual samples for within-person comparisons, and use more challenging reasoning tasks such as polarity-varied Wason stimuli to distinguish matching bias from confirmation bias. Longitudinal designs tracking models across updates, process-tracing studies examining step-by-step reasoning, more representative human samples, and systematic API versus interface comparisons would collectively advance understanding of how cognitive biases manifest across languages and model generations.

## 6 Conclusion

This study examined two classic cognitive biases and cross-language reasoning performance across three Large Language Models (ChatGPT, Gemini, and Claude) in three languages (English, Russian, and Hebrew) under “reasoning-only” instructions.

The analysis identified a possible “cognitive gap” in contemporary Large Language Models (LLMs): in logic-pure tasks (e.g., the Wason Selection Task), the models typically followed the rules consistently and independently of human error patterns. However, GPT’s performance in Russian indicated that even logical reasoning can be language-dependent, providing ground for future research. In contrast, when confronted with heuristic tasks (Original and Modified Word Frequency Tests), models exhibited consistent language-conditioned variability, similar to those observed in human participants. Taken together, these findings suggest the cognitive gap is not absolute, but may depend both on the task type and linguistic context.

Additionally, and perhaps more fundamentally, all three LLMs demonstrated significant cross-linguistic variations in their core reasoning accuracy, establishing that language choice constitutes a decisive factor in performance outcomes. Rather than demonstrating uniform execution across languages or predictable cross-language hierarchies, the models exhibited complex, task-dependent performance variations indicative of language-specific processing mechanisms.

This study challenges both assumptions of optimal prompt languages and expectations of uniform multilingual performance, suggesting that model-internal processing interacts with linguistic structures in unpredictable ways. Bias expression and reasoning performance in Large Language Models seem to depend on language-specific processing mechanisms rather than universal patterns.

From a technology management perspective, the research points to important considerations for organizational leaders looking to implement AI systems across global operations. Decision-makers in multinational corporations, particularly in high-stakes sectors such as finance and healthcare, should be aware that AI advisory systems may exhibit fundamentally different cognitive bias patterns depending on the input language. This linguistic variability adds complexity to risk assessment, suggesting that organizations could benefit from developing new governance structures and evaluation protocols that incorporate cross-linguistic testing of AI systems, including language-specific validation procedures and multilingual bias-monitoring frameworks. Consequently, this research offers useful empirical evidence for technology managers seeking to balance the transformative potential of LLMs with responsible deployment practices that acknowledge their complex and language-dependent behavioral characteristics.

For everyday practical use, these findings point to the importance of critical thinking when using Large Language Models, particularly in decision-making contexts or across languages. Users should be aware that the same model may exhibit varying cognitive bias-like patterns and reasoning capabilities, depending on the language of interaction or the task at hand. Future developments in multilingual AI systems must therefore account for these variations to ensure reliable performance across diverse linguistic contexts. In conclusion, this work demonstrates that while Large Language Models approach and even surpass human cognitive capacity in specific reasoning domains, they simultaneously exhibit systematic cognitive vulnerabilities that manifest unpredictably across linguistic contexts. Recognizing and understanding these language-dependent blind spots represents a critical advancement toward developing more reliable AI technologies and establishing responsible governance frameworks that account for the complex, non-uniform nature of artificial intelligence reasoning capabilities.

## Data Availability

The datasets presented in this study can be found in online repositories. The names of the repository/repositories and accession number(s) can be found at https://github.com/KatyaGrander/Cross-Linguistic-Patterns-of-Cognitive-Biases-in-LLMs-A-Comparative-Study.
