# Correction: Cross-linguistic patterns of cognitive biases in large language models: a comparative study in English, Hebrew, and Russian

**DOI:** 10.3389/frai.2026.1925443

**Published:** 2026-08-07

**Authors:** Katya Grander, Victoria Mosub, Sigal Kordova

**Affiliations:** 1 Faculty of Industrial Engineering and Technology Management, Holon Institute of Technology, Holon, Israel; 2 Department of Industrial Engineering and Management, Ariel University, Ariel, Israel

**Keywords:** large language models, cognitive biases in LLMs, cross-linguistic LLM performance, machine psychology, LLM-human reasoning comparison, multilingual AI evaluation

Author Katya Grander was erroneously assigned as corresponding author. The correct corresponding author is Sigal Kordova.

In the first paragraph of section *3.3 Response correctness criteria* Hebrew translation for “yes” and “no” is incorrectly displayed backwards as ‘ןכ” and “ אל”. It should be displayed ‘כן” and “ לא”, similar to the rest of the manuscript. The correct paragraph should read:

To establish the foundation for “correct”/“incorrect” responses, regarding the frequency of the words “Yes” and “No” in the English language, “Да” and “Нет” in Russian, and “כן” and “ לא” in Hebrew, three text corpora were analyzed—one for each language:

Section *4.4 LLMs' cross-linguistic performance*, authors found a human error in result description. The error is purely technical and does not affect the outcome of the study, since the correct results are displayed and addressed otherwise throughout. The correction is requested to avoid any confusion.

In section 4.4.1.3 Modified Wason selection task, references to the term “English” and “Russian” were interchanged, as well as the term “better” for “worse”. The correct paragraphs should read:

Following significant Chi-Square results, *post-hoc* pairwise comparisons were conducted to identify which LLMs differed across source languages. Bonferroni corrections were applied for multiple comparisons.

For the Modified Word Frequency Test all models showed a strong language-dependent pattern. ChatGPT achieved significantly higher accuracy in Hebrew and Russian than in English (*p* < 0.001), with no difference between Hebrew and Russian (*p* = 1.000). Claude showed significantly lower performance in English than in both Russian and Hebrew (both *p* =.011), while Russian and Hebrew did not differ significantly (*p* = 1.000).

Gemini displayed significantly higher accuracy in Hebrew than in English and Russian (both *p* < 0.001), whereas English and Russian did not differ significantly (*p* = 1.000).

In the Original Word Frequency Test, *post-hoc* comparisons showed that ChatGPT performed significantly worse in English and Hebrew than in Russian (both *p* < 0.001), with a small but statistically significant difference between English and Hebrew (*p* = 0.001). Claude showed significantly lower performance in Russian than in both English and Hebrew (both *p* = 0.011), whereas no significant difference was found between English and Hebrew (*p* = 1.000). Gemini demonstrated a significantly lower performance in Russian than in both English (*p* = 0.004) and Hebrew (*p* < 0.001). No significant difference was observed between English and Hebrew performance (*p* = 0.726).

The original version of this article has been updated.

